# Inhibition of SDE2 promotes autophagy-dependent ferroptosis in multiple myeloma

**DOI:** 10.1016/j.redox.2026.104007

**Published:** 2026-01-16

**Authors:** Liang Xia, Jing Bao, Xiao-wen Chen, Yu-Chen Zhao, Xiang Wang, Yu Zheng

**Affiliations:** a Department of Hematology, The First Affiliated Hospital, Anhui Medical University, Hefei, Anhui, 230031, China; b Shanghai Institute of Hematology, State Key Laboratory of Medical Genomics, National Research Center for Translational Medicine at Shanghai, Ruijin Hospital Affiliated to Shanghai Jiao Tong University School of Medicine, Shanghai, 200025, China

**Keywords:** Multiple myeloma, SDE2, ATG5, Autophagy, Ferroptosis, Ubiquitination

## Abstract

**Background:**

Multiple myeloma (MM) is an incurable plasma cell malignancy with high relapse rate. Recent studies have implicated dysregulated autophagy and ferroptosis in MM progression; however, the molecular links remain elusive. This study investigated the role of SDE2, a ubiquitin-like protein overexpressed in MM, in modulating autophagy-ferroptosis crosstalk via ATG5 degradation with the aim of identifying novel therapeutic targets.

**Methods:**

Using bioinformatic analysis of TCGA data, we identified SDE2 as a prognostic marker in MM. Functional validation included Western blot, co-immunoprecipitation, and ubiquitination assays in MM cell lines (H929, RPMI8226, OPM-2, and KMS-11) and patient-derived samples. Transwell migration, soft agar colony formation, and flow cytometry were used to assess cellular phenotypes. *In vivo* efficacy was tested using xenograft models.

**Results:**

SDE2 overexpression correlates with poor MM prognosis and promotes tumor cell survival, migration, and proliferation. Mechanistically, SDE2 binds to ATG5, facilitating K48-linked ubiquitination and proteasomal degradation, thereby suppressing autophagy and ferroptosis. Knockdown of SDE2 restored ATG5 levels, reactivated autophagy, and sensitized MM cells to ferroptosis. Combined SDE2 silencing and pharmacological ATG5/7 activation (Antitumor agent-82) synergistically suppressed tumor growth *in vitro* and *in vivo*.

**Conclusion:**

The SDE2-ATG5 axis serves as a critical regulator of the autophagy-ferroptosis crosstalk in MM. Targeting SDE2 restores ATG5-dependent autophagy, activates ferroptosis, and inhibits tumor growth. These findings suggest a novel therapeutic strategy that combines SDE2 inhibitors with autophagy agonists, potentially offering clinical benefits in MM treatment. This study provides further insight into autophagy-dependent ferroptosis in other malignancies.

## Graphical abstract

**Figure:**
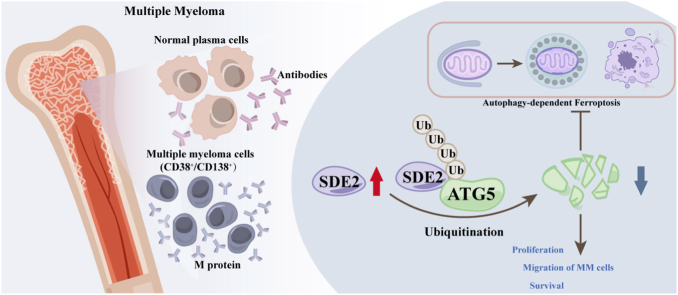

## 1 Introduction

Multiple myeloma (MM), a malignancy of plasma cells originating from the bone marrow, continues to pose significant challenges in the field of hematological oncology [1]. Despite advances in chemotherapy, immunotherapy, and targeted treatment, MM is characterized by high recurrence rates and poor long-term survival in many patients [2]. The pathogenesis of MM is driven by genetic mutations and alterations in the bone marrow microenvironment, leading to uncontrolled plasma cell proliferation, production of monoclonal immunoglobulins, and severe complications including bone lesions, renal dysfunction, and immune suppression [3,4]. The increasing prevalence of MM, coupled with its complex pathophysiology, underscores the urgent need for deeper mechanistic insights and development of novel therapeutic strategies.

Although proteasome inhibitors, immunomodulatory drugs, and monoclonal antibodies have significantly improved clinical outcomes, therapeutic resistance and disease relapse remain pervasive challenges [[5], [6], [7]]. The molecular mechanisms underlying MM progression, particularly the roles of autophagy and ubiquitination, are not fully understood, limiting the development of durable therapies [8,9]. Tumor heterogeneity, marked by variable gene expression profiles and diverse cellular phenotypes, further complicates the design of effective and personalized treatment regimens [10]. Moreover, the intricate interplay between MM cells and the bone marrow microenvironment plays a central role in both disease progression and resistance to existing therapies, creating significant barriers to long-term success.

Recent studies have highlighted the importance of autophagy, a cellular recycling mechanism, and ferroptosis, an iron-dependent form of regulated cell death, which is a pivotal process in cancer biology [[11], [12], [13], [14]]. Autophagy has a dual role in MM, functioning as a survival mechanism in certain contexts while promoting cell death under others [[15], [16], [17]]. Similarly, ferroptosis has emerged as a promising therapeutic strategy because of its ability to overcome apoptotic resistance in MM cells [18,19]. Ubiquitination, a post-translational modification that regulates protein stability, has been implicated in modulating both the autophagy and ferroptosis pathways [[20], [21], [22], [23]], suggesting that it may serve as a critical regulatory hub in MM progression. However, the molecular crosstalk between these pathways and their precise contributions to MM pathogenesis remain poorly defined.

Among the regulators of ubiquitin-mediated signaling, SDE2, a ubiquitin-like protein primarily involved in DNA replication stress responses and proteasomal degradation [24,25], has emerged as a candidate of interest. Although SDE2 has been studied in genome maintenance and cell cycle control, recent datasets suggest it is aberrantly expressed in several cancers [26,27]. Yet, its biological role in MM remains elusive. Notably, ATG5, a key regulator of autophagosome formation, is known to be tightly controlled by ubiquitination [28]. Given that autophagy can either promote or inhibit ferroptosis depending on the cellular context, it is plausible that SDE2 may influence ferroptosis responses in MM by modulating ATG5 stability and thereby altering autophagic activity. Despite this potential mechanistic link, the functional relevance of the SDE2–ATG5–ferroptosis axis in MM has yet to be investigated.

To address this gap, we propose to investigate how SDE2-driven regulation of ATG5 contributes to MM progression through the modulation of autophagy and ferroptosis. Specifically, we hypothesize that SDE2 facilitates tumor progression by enhancing the ubiquitin-mediated degradation of ATG5, which in turn suppresses autophagy and dampens ferroptosis. Targeting SDE2 may therefore represent a novel therapeutic strategy to re-establish autophagy–ferroptosis crosstalk and inhibit MM development.

Accordingly, the objectives of this study are: (1) to elucidate the mechanistic role of the SDE2–ATG5 axis in regulating autophagy and ferroptosis in MM; (2) to assess the therapeutic potential of SDE2 inhibition, alone or in combination with pharmacological autophagy inducers; and (3) to evaluate the *in vitro* and *in vivo* impact of SDE2 inhibition on MM progression.

## 2 Materials and methods

### 2.1 Ethics statement

All human samples were obtained with informed consent under protocols approved by the Institutional Review Board of Ruijin Hospital Affiliated to Shanghai Jiao Tong University School of Medicine. Animal experiments were conducted in accordance with the guidelines of the Animal Care and Use Committee of Ruijin Hospital Affiliated to Shanghai Jiao Tong University School of Medicine.

### 2.2 Patient samples

Bone marrow aspirates were collected from patients with MM and healthy donors. CD38^+^/CD138^+^ plasma cells (tumor cells, T) and non-tumor bone marrow cells (N) were isolated using magnetic-activated cell sorting (MACS; Miltenyi Biotec).

### 2.3 Cell lines and culture

Multiple myeloma cell lines—H929, RPMI 8226, OPM-2, and KMS-11—and HEK293T cells were obtained from the Shanghai Institute of Life Sciences (Shanghai, China). The MM cell lines were cultured in RPMI-1640 medium supplemented with 10 % fetal bovine serum (FBS) and 1 % penicillin-streptomycin (P/S). Specifically, for H929 cells, 0.05 mM β-mercaptoethanol was added to the culture medium. HEK293T cells were maintained in Dulbecco's modified Eagle's medium (DMEM) supplemented with 10 % FBS, 6 mM l-glutamine, and 1 % P/S. All cells were incubated at 37 °C in a humidified atmosphere containing 5 % CO_2_. Peripheral blood mononuclear cells (PBMCs) from healthy donors were isolated using Ficoll-Paque density gradient centrifugation (GE Healthcare).

### 2.4 Plasmid construction and transfection

**shRNA Design and Synthesis:** Two shRNAs targeting SDE2 (**shSDE2-1**: 5′-gaggttcggg tagtcggtga TTCAAGAGA tcaccgacta cccgaacctc-3’; **shSDE2-2**: 5′-catggcggag gccgcggcgc TTCAAGAGA gcgccgcggc ctccgccatg-3′) and one targeting ATG5 (**shATG5**: 5′-atgacagatg acaaagatgt TTCAAGAGA acatctttgt catctgtcat-3′) were synthesized by GenePharma (Suzhou, China). A non-targeting control shRNA was also synthesized for use as a negative control.

**SDE2 Overexpression Plasmids:** Wild-type SDE2 (NM_152608.4) and its mutants were synthesized by GenePharma.

**ATG5 Overexpression Plasmids:** ATG5 (NM_001286106.2) was cloned into the pcDNA3.1 vector and verified by sequencing. **Ubiquitination Assay Plasmids:** HA-tagged ubiquitin plasmids (wild-type: #17608; K48-linked: #17605; K63-linked: #17606) were obtained from Addgene.

**Transfection and Lentiviral Transduction:** Plasmid transfections were performed using Lipofectamine 3000 (Thermo Fisher) according to the manufacturer's instructions. Lentiviral packaging was performed by GenePharma in HEK293T cells, and transductions were conducted using Lipofectamine 3000. Stable cell lines were selected with 2 μg/mL puromycin (Sigma-Aldrich) for 7–10 days.

### 2.5 Bioinformatics analysis

The gene expression datasets (GSE136337 and GSE24080) were retrieved from the Gene Expression Omnibus (GEO) database. Differential expression analysis of SDE2 and ATG5 was conducted using limma (Bioconductor), with significance thresholds set at adjusted P-value <0.05, and |log_2_ fold change| > 1. Survival analysis was performed using Kaplan-Meier estimates and log-rank tests.

### 2.6 Quantitative real-time PCR (qRT-PCR)

Total RNA was extracted from cells using TRIzol reagent (Invitrogen) and reverse-transcribed into cDNA using a High-Capacity cDNA Reverse Transcription Kit (4368814, Thermo Fisher) according to the manufacturer's instructions. qRT-PCR was performed using SYBR Green Master Mix on QuantStudio 6 (Thermo Fisher) with GAPDH as the internal control. Primer sequences were as follows: **SDE2:** Forward 5′- TGCACCGTCCGGGATTTTATC-3′, Reverse 5′- CTGCACTGTGTCACTGGTGTT-3'

**ATG5:** Forward 5′-AAAGATGTGCTTCGAGATGTGT-3′, Reverse 5′-CACTTTGTCAGTTACCAACGTCA-3'

**GAPDH:** Forward 5′-GGAGCGAGATCCCTCCAAAAT -3′, Reverse 5′-GGCTGTTGTCATACTTCTCATGG -3'

### 2.7 Western blot analysis

Proteins were extracted using RIPA lysis buffer (Beyotime) containing protease inhibitors (Roche). Lysates were resolved by SDS-PAGE, transferred to PVDF membranes (Millipore), and probed with antibodies against SDE2 (PA5-46779, Thermo Fisher), ATG5 (#2630, Cell Signaling Technology), LC3 (sc-271625, Santa Cruz), P62 (sc-28359, Santa Cruz), and GAPDH (ab8245, Abcam).

### 2.8 Co-immunoprecipitation (Co-IP)

For crosslinking experiments, cell lysates were treated with 1 mM EDC (Thermo Fisher) for 30 min at room temperature before proceeding to immunoprecipitation. Subsequently, the lysates were incubated with anti-SDE2, anti-ATG5, anti-Flag, or anti-Myc antibodies overnight at 4 °C to allow antibody-antigen binding. Protein complexes were then captured using Protein A/G agarose beads (Thermo Fisher). After extensive washing with lysis buffer to remove non-specific interactions, the precipitated complexes were eluted and analyzed by Western blotting.

### 2.9 Functional assays

**Transwell Migration**: Cells (1 × 10^5^) in serum-free medium were seeded into Transwell chambers (Corning). After 24 h incubation, migrated cells were stained with 0.1 % crystal violet.

**Soft Agar Colony Formation**: Cells (5 × 10^3^) in 0.3 % agar (Sigma) were layered over 0.6 % agar in 6-well plates. Colonies were counted after 14 days.

**Flow Cytometry**: Apoptosis was assessed using PI/CFDA co-staining (BD Biosciences). Data were acquired on the BD FACSCanto II and analyzed with FlowJo v10.

### 2.10 Autophagy and ferroptosis evaluation

**Dual-Fluorescence LC3 Reporter**: Lentiviral mRFP-GFP-LC3 (GeneChem) was transduced into cells. The autophagic flux was quantified by confocal microscope (Zeiss LSM 880).

**Lipid Peroxidation**: BODIPY 581/591 C11 (Thermo Fisher) was used to measure lipid ROS levels.

**Fe^2+^ and MDA Assays**: Intracellular Fe^2+^ was quantified using an Iron Assay Kit (Abcam), and malondialdehyde (MDA) levels were measured using a thiobarbituric acid assay (Sigma).

### 2.11 *In vivo* xenograft model

Male NOD/SCID mice (6–8 weeks old, Vital River) were subcutaneously injected with 2 × 10^6^ MM cells. Tumor volume was measured every 7 days using calipers and tumor volume was calculated as (length × width [2])/2. Mice were treated with Antitumor agent-82 (5 mg/kg, i.p.) or vehicle. At the end of the study, mice were euthanized and tumors were excised for histological analysis (H&E, Ki67 IHC) and WB analysis. All animal experiments were approved by the Institutional Animal Care and Use Committee.

### 2.12 Statistical analysis

Data are presented as mean ± SD from three independent experiments. Differences were analyzed by Student's t-test (two groups) or one-way ANOVA (multiple groups) using GraphPad Prism v9.0. ∗P < 0.05 was considered significant.

## 3 Results

### 3.1 SDE2 overexpression as a prognostic indicator in multiple myeloma

**Fig. 1:**
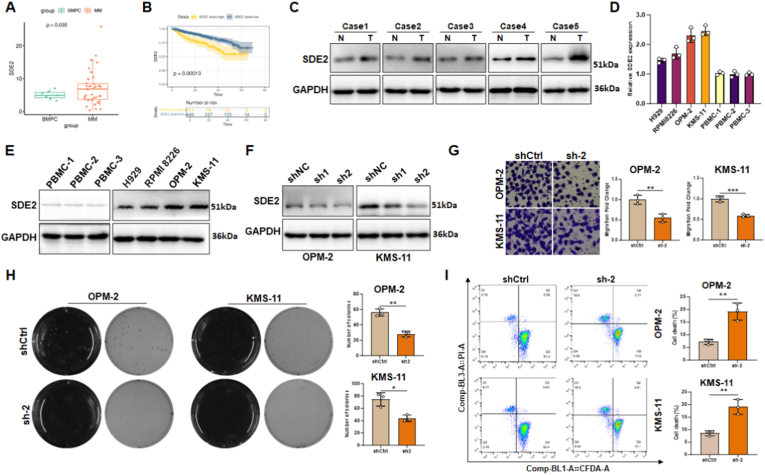
Functional characterization of SDE2 in MM cell lines. (A) Box plot depicting significantly elevated SDE2 expression in plasma cells from MM patients compared to healthy controls, based on TCGA data. (B) Kaplan-Meier survival curves illustrating a significant difference in overall survival between MM patients with high and low SDE2 expression, derived from TCGA data. (C) Western blot analysis showing SDE2 expression in malignant cells (T) and non-malignant bone marrow cells (N) isolated from the peripheral blood of MM patients. (D) qPCR analysis of SDE2 mRNA levels in MM cell lines (H929, RPMI 8226, OPM-2, and KMS-11) compared to PBMCs from healthy donors. (E) Western blot analysis of SDE2 protein levels in MM cell lines and healthy donor PBMCs. (F) Western blot validation of SDE2 knockdown efficiency using two shRNAs (sh1 and sh2) in OPM-2 and KMS-11 cells. (G) Crystal violet staining of Transwell migration assays comparing the migratory potential of OPM-2 and KMS-11 cells with or without SDE2 knockdown (left). The bar graph quantifies fold changes in migration (right). (H) Representative soft agar colony formation assay images under epi-illumination and transmitted light (left). The bar graph quantifies the number of colonies formed (right). Images were captured using a ChemiDoc MP system. (I) Flow cytometry analysis of PI/CFDA-stained OPM-2 and KMS-11 cells with or without SDE2 knockdown. Quadrant 2 (Q2) indicates dead cells (PI-positive/CFDA-negative), and quadrant 4 (Q4) indicates live cells (CFDA-positive/PI-negative) (left). The bar graph displays the percentage of dead cells (right). ∗P < 0.05; ∗∗P < 0.01; ∗∗∗P < 0.001.

Bioinformatics analysis utilizing the TCGA database revealed a significant upregulation of SDE2 expression in the bone marrow of patients with multiple myeloma (Fig. 1A). Moreover, patients with elevated SDE2 levels experienced a more rapid decline in survival rates (Fig. 1B), indicating that SDE2 overexpression serves as a risk factor contributing to the onset and progression of MM.

To corroborate these findings, we isolated bone marrow-derived plasma cells (CD38^+^/CD138^+^) from clinical MM patient samples and compared their SDE2 expression levels to those in non-tumorous bone marrow cells. Western blot analysis demonstrated a marked increase in SDE2 expression in MM cells relative to non-tumorous counterparts (Fig. 1C). Similarly, peripheral blood mononuclear cells (PBMCs) from healthy volunteers and various human-derived MM cell lines (H929, RPMI 8226, OPM-2, and KMS-11) were assessed for SDE2 expression. The results showed consistent SDE2 expression in PBMCs from different healthy donors, whereas MM cell lines exhibited heterogeneous yet significantly elevated SDE2 levels compared to healthy PBMCs (Fig. 1D and E).

Recognizing that SDE2 is not a classical oncogene, we investigated whether its elevated expression in MM promotes disease progression or whether it represents an adaptive response. We designed and introduced two shRNAs targeting SDE2 (sh1 and sh2) into the OPM-2 and KMS-11 MM cell lines. The sh2 construct, which demonstrated more effective silencing, was selected for subsequent SDE2 knockdown experiments (Fig. 1F). Conversely, SDE2 was overexpressed in the H929 and RPMI 8226 cell lines (Fig. S1A).

Transwell migration assays revealed a significant reduction in MM cell migration following SDE2 knockdown (Fig. 1G), whereas SDE2 overexpression substantially increased migration (Fig. S1B). Similarly, soft agar colony formation assays indicated a notable decrease in MM cell proliferation following SDE2 knockdown (Fig. 1H), whereas SDE2 overexpression markedly enhanced the cell proliferation (Fig. S1C). To evaluate cell viability and apoptosis, dual staining with propidium iodide (PI) and carboxyfluorescein diacetate (CFDA) was performed. Flow cytometry analysis revealed a significant increase in the proportion of dead cells following SDE2 knockdown (Fig. 1I). In contrast, SDE2 overexpression significantly decreased the proportion of dead cells (Fig. S1D).

In summary, our data identified and validated that SDE2 is a crucial promoter of MM initiation and progression, playing a key role in MM cell survival, proliferation, and migration.

### 3.2 SDE2 negatively regulates ATG5 in multiple myeloma cells

**Fig. 2:**
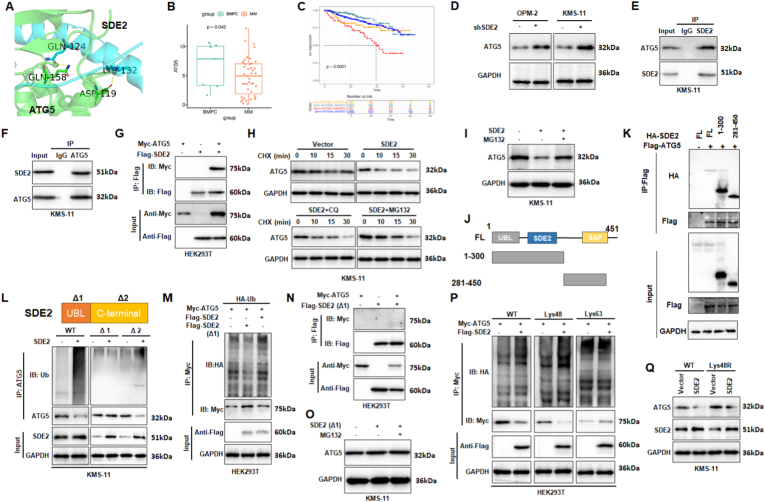
Molecular interaction between SDE2 and ATG5. (A) Molecular docking prediction illustrating the interaction between ATG5 and SDE2. (B) Box plot showing significantly elevated ATG5 expression in plasma cells from MM patients compared to healthy controls. Data were obtained from the TCGA database. (C) Kaplan–Meier survival analysis of multiple myeloma (MM) patients stratified by combined expression levels of ATG5 and SDE2. (D) Western blot analysis demonstrating the effect of SDE2 knockdown on ATG5 protein levels in OPM-2 and KMS-11 cells. (E–F) Co-immunoprecipitation (Co-IP) assays in KMS-11 cells using antibodies targeting SDE2 to pull down ATG5 (E) and antibodies targeting ATG5 to pull down SDE2 (F), confirming a direct interaction between the two proteins. (G) HEK 293T cells were co-transfected with Myc-tagged ATG5 and Flag-tagged SDE2. Immunoprecipitation using anti-Flag antibodies was followed by immunoblotting with anti-Myc (ATG5) and anti-Flag (SDE2) antibodies, validating the interaction between exogenous SDE2 and ATG5. (H) Western blot analysis of ATG5 degradation in SDE2-overexpressing cells treated with the protein synthesis inhibitor cycloheximide (CHX, 10 μg/mL) in the presence of chloroquine (CQ) or MG132. (I) Western blot analysis showing that treatment with MG132 rescues ATG5 degradation in SDE2-overexpressing cells. (J) Schematic representation of full-length and truncation constructs of SDE2. (K) Co-immunoprecipitation of HA-SDE2 variants with Flag-tagged ATG5 in HEK293T cells. Only full-length and 1–300 aa fragment of SDE2 retained the ability to bind ATG5. (L) Cell lysates from SDE2-overexpressing cells (wild-type, Δ1, and Δ2 mutants) were immunoprecipitated with anti-ATG5 antibodies and immunoblotted with anti-Ub and anti-ATG5 antibodies to assess ATG5 ubiquitination levels. (M) HEK 293T cells were co-transfected with HA-tagged ubiquitin (Ub), Myc-tagged ATG5, and Flag-tagged SDE2 (wild-type and Δ1 mutant). Immunoprecipitation using anti-Myc antibodies was followed by immunoblotting with anti-HA and anti-Myc antibodies, demonstrating that the SDE2 UBL domain mediates ATG5 ubiquitination. (N) Co-IP analysis of the interaction between ATG5 and the SDE2-Δ1 mutant. HEK293T cells were co-transfected with Myc-tagged ATG5 and Flag-tagged SDE2-Δ1 plasmids as indicated. Cell lysates were immunoprecipitated with anti-Flag antibody, followed by immunoblotting with anti-Myc and anti-Flag antibodies. Input blots confirmed protein expression levels. (O) Fig. 4M. SDE2-Δ1 fails to promote ATG5 degradation in KMS-11 cells. Cells were transfected with SDE2-Δ1 and treated with or without the proteasome inhibitor MG132 (10 μM, 6 h). (P) HEK 293T cells were co-transfected with Myc-tagged ATG5, Flag-tagged SDE2, and HA-tagged ubiquitin constructs (wild-type, Lys48-only, or Lys63-only). Immunoprecipitation using anti-Myc antibodies was followed by immunoblotting with anti-HA and anti-Myc antibodies, confirming that SDE2 facilitates Lys48-linked ubiquitination of ATG5. (Q) Western blot analysis showing ATG5 and SDE2 levels in control and SDE2-overexpressing KMS-11 cells co-expressing wild-type ubiquitin (Ub WT) or ubiquitin with a Lys48-to-Arg mutation (Ub Lys48R) after 72 h of culture. ∗P < 0.05; ∗∗P < 0.01; ∗∗∗P < 0.001.

Molecular docking analysis identified a potential binding interface between ATG5 and SDE2, suggesting the formation of hydrogen bonds (Fig. 2A). Clinical data further demonstrated a significant reduction in ATG5 expression in the bone marrow cells isolated from patients with MM (Fig. 2B). Kaplan–Meier survival analysis further revealed that patients with low ATG5 and high SDE2 expression exhibited the poorest prognosis, whereas those with low expression of both genes had the most favorable outcomes (Fig. 2C). These findings support a functional interplay between SDE2 and ATG5, and raise the possibility that SDE2 may directly interact with and negatively regulate ATG5 during MM progression.

To investigate this relationship, we assessed ATG5 expression in OPM-2 and KMS-11 cells, with and without SDE2 knockdown. The results indicate an increase in ATG5 levels following SDE2 knockdown (Fig. 2D). Co-immunoprecipitation (Co-IP) experiments, utilizing EDC cross-linking, confirmed a direct interaction between SDE2 and ATG5. Immunoprecipitation targeting SDE2 successfully pulled down ATG5, and vice versa (Fig. 2E–F). Additionally, co-transfection of Myc-tagged ATG5 and Flag-tagged SDE2 in HEK293T cells, followed by co-IP with an anti-Flag antibody, detected Myc-positive bands, corroborating the interaction between endogenous and exogenous ATG5 and SDE2 (Fig. 2G).

To elucidate the mechanism underlying ATG5 downregulation, we employed cycloheximide (CHX) to inhibit protein synthesis, thereby allowing the measurement of protein degradation rates. In KMS-11 cells overexpressing SDE2, ATG5 degradation was significantly accelerated compared to that in control cells. Considering that proteins are primarily degraded via autophagy or the ubiquitin-proteasome system, we treated SDE2-overexpressing cells with chloroquine (CQ) or MG132, respectively, to inhibit autophagy or the proteasome. CQ treatment did not prevent SDE2-induced acceleration of ATG5 degradation, whereas MG132 effectively mitigated this effect (Fig. 2H). Further validation showed that MG132 treatment rescued ATG5 reduction caused by SDE2 overexpression, providing strong evidence that SDE2 promotes ATG5 degradation through the ubiquitin-proteasome pathway (Fig. 2I).

We next aimed to dissect the structural basis of this interaction. Truncation constructs of SDE2 were designed (Fig. 2J), and Co-IP experiments revealed that the N-terminal fragment (1–300 aa), which includes the UBL and SDE2 domains, exhibited stronger binding to ATG5 than the C-terminal fragment (281–450 aa) (Fig. 2K). Given that the N-terminal region of SDE2 harbors a conserved KKGKK motif within its ubiquitin-like (UBL) domain, it is potentially cleaved by deubiquitinating enzymes, resulting in two functional fragments: the Sde2-UBL and Sde2-C terminal portions. This proteolytic processing suggests that the UBL-containing N-terminus might be functionally critical for mediating the interaction with and ubiquitination of ATG5. To further validate the importance of the UBL domain, we constructed a UBL deletion mutant (Δ1) and a C-terminal deletion mutant (Δ2) (Fig. 2L). Co-IP and ubiquitination assays in KMS-11 cells revealed that while wild-type (WT) and Δ2 SDE2 maintained ATG5 ubiquitination and degradation ability, Δ1 SDE2 lost this capacity (Fig. 2L). The loss of function was also confirmed in ubiquitination assays using HA-tagged ubiquitin (Fig. 2M), and binding between ATG5 and SDE2-Δ1 was markedly diminished (Fig. 2N). Moreover, MG132 treatment had no further impact on ATG5 expression in the presence of SDE2 (Δ1), reinforcing that the UBL domain is required for the proteasome-dependent degradation of ATG5 (Fig. 2O).

To assess the specificity of SDE2 ubiquitination, we introduced various recombinant ubiquitin constructs into HEK293T cells, including HA-Ub-WT, HA-Ub-Lys48-only, and HA-Ub-Lys63-only. These findings indicated that Lys48-linked ubiquitin, similar to wild-type ubiquitin, increased ubiquitin binding to ATG5 and promoted its degradation (Fig. 2P). In KMS-11 cells, ubiquitin with a Lys48-to-Arg mutation failed to facilitate the SDE2-induced ATG5 degradation (Fig. 2Q).

In summary, SDE2 interacts with ATG5 and its UBL domain promotes Lys48-linked ubiquitination of ATG5, leading to its degradation via the ubiquitin-proteasome pathway.

### 3.3 Inhibition of SDE2 enhances autophagy in multiple myeloma cells

**Fig. 3:**
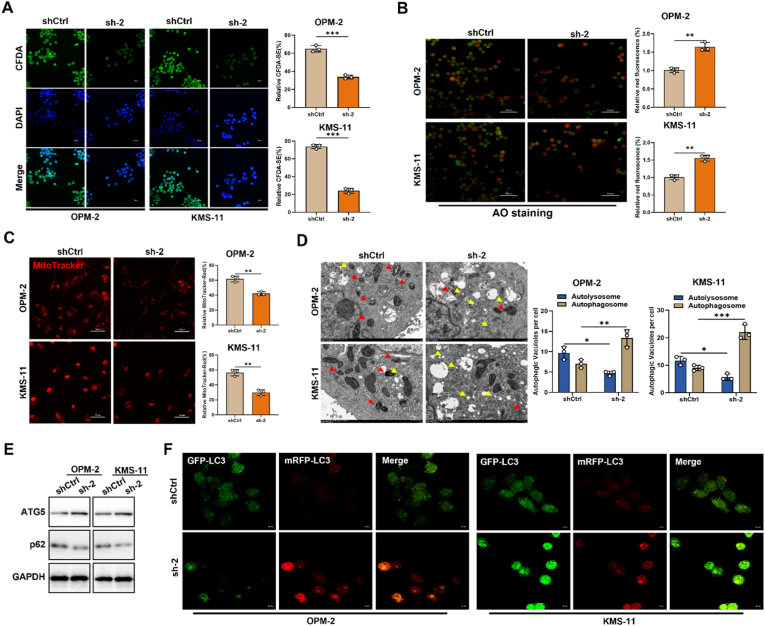
Impact of SDE2 knockdown on cellular metabolism and autophagy in MM cells. (A) Fluorescence imaging of CFDA/DAPI-stained OPM-2 and KMS-11 cells with or without SDE2 knockdown (left). The bar graph quantifies relative fluorescence intensity (right). (B) Fluorescence imaging of acridine orange-stained OPM-2 and KMS-11 cells with or without SDE2 knockdown (left). The bar graph quantifies relative fluorescence intensity (right). (C) MitoTracker staining to evaluate mitochondrial activity in OPM-2 and KMS-11 cells with or without SDE2 knockdown. (D) Transmission electron microscopy (TEM) images showing autophagosomes (indicated by red arrows) and autolysosomes (indicated by yellow arrows) in OPM-2 and KMS-11 cells with or without SDE2 knockdown. (E) Western blot analysis of p62 and ATG5 expression levels in OPM-2 and KMS-11 cells with or without SDE2 knockdown. (F) mRFP-GFP-LC3 dual fluorescence labeling of OPM-2 and KMS-11 cells with or without SDE2 knockdown, visualizing autophagosomes (yellow puncta: red + green) and autolysosomes (red-only puncta). The bar graphs quantify the total number of puncta and the relative proportion of autophagosomes to autolysosomes. ∗P < 0.05; ∗∗P < 0.01; ∗∗∗P < 0.001.

Fluorescent imaging using CFDA-DAPI co-staining demonstrated that SDE2 knockdown significantly reduced MM cell viability (Fig. 3A). Conversely, the overexpression of SDE2 markedly increased cell survival (Fig. S1E). Acridine orange staining further corroborated these findings, revealing a substantial increase in red fluorescence, which is indicative of cell death, following SDE2 knockdown (Fig. 3B).

MitoTracker staining, which assesses mitochondrial quantity, membrane integrity, and activity, revealed suppressed mitochondrial function upon SDE2 downregulation (Fig. 3C). In contrast, SDE2 overexpression enhanced the mitochondrial activity (Fig. S1F). Given ATG5's essential role in autophagy, we hypothesized that the observed changes in mitochondrial function might be linked to autophagy modulation.

Transmission electron microscopy (TEM) analysis revealed an increase in autolysosomes and a decrease in autophagosomes, suggesting accelerated fusion between autophagosomes and lysosomes, thereby enhancing the autophagic process (Fig. 3D). Consistent with this observation, the levels of P62—a key autophagy substrate inversely correlated with autophagic activity, were significantly reduced in OPM-2 and KMS-11 cells upon SDE2 knockdown (Fig. 3E). Conversely, SDE2 overexpression in H929 and RPMI 8226 cells led to a significant increase in P62 levels (Fig. S1G), further supporting SDE2's role in autophagy regulation.

To further assess autophagic flux, we employed an mRFP-GFP-LC3 dual-fluorescence system. In this assay, the mRFP tag remained stable under acidic conditions, whereas the GFP tag was quenched, allowing differentiation between autophagosomes (yellow puncta) and autolysosomes (red-only puncta). Our results showed that ATG5 upregulation significantly increased overall fluorescence intensity, particularly red fluorescence, indicating accelerated autolysosome formation and enhanced autophagic turnover (Fig. 3F).

Notably, while TEM analysis showed a significant reduction in autophagosomes in KMS-11 cells after SDE2 knockdown, the mRFP-GFP-LC3 system showed an increase in green fluorescence intensity. This discrepancy may be due to morphological counting bias and elevated LC3 expression levels. In contrast, ATG5 downregulation in H929 and RPMI 8226 cells led to decreased fluorescence intensity, particularly red fluorescence, suggesting impaired autophagosome-lysosome fusion and overall suppression of autophagic flux (Fig. S1H).

Collectively, these findings indicate that SDE2 negatively regulates autophagy in MM cells, and its inhibition enhances autophagic activity, potentially contributing to reduced cell viability.

### 3.4 SDE2 negatively regulates the ferroptosis pathway in multiple myeloma cells

Although we have established a negative correlation between SDE2 and autophagy, the precise mechanism by which SDE2 influences MM cell viability remains to be fully elucidated. Previous studies have demonstrated that excessive autophagy, particularly ferritinophagy, can disrupt cellular iron homeostasis, leading to Fe^2+^ accumulation and subsequent ferroptosis. Specifically, the Fenton reaction, driven by Fe^2+^, generates excessive reactive oxygen species (ROS), which oxidize membrane lipids into lipid peroxides, thereby impairing cellular structures and functions, ultimately causing cell death.

**Fig. 4:**
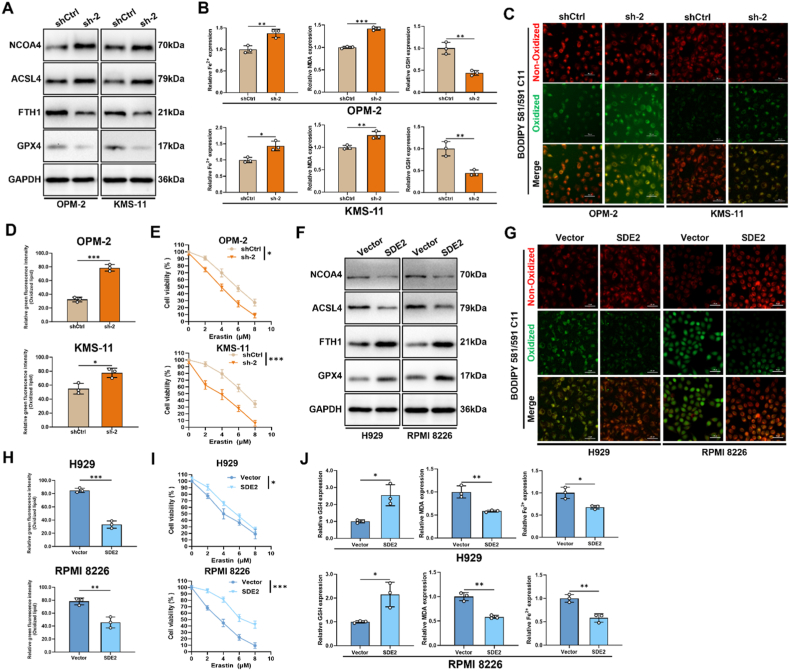
Role of SDE2 in regulating ferroptosis in MM cells. (A) Western blot analysis of ferroptosis-related proteins (NCOA4, ACSL4, FTH1, and GPX4) in OPM-2 and KMS-11 cells with or without SDE2 knockdown. (B) Biochemical analysis of ferroptosis-related markers (Fe^2+^, MDA, and GSH) in OPM-2 and KMS-11 cells with or without SDE2 knockdown. (C) BODIPY C11 probe staining visualizing intracellular reactive oxygen species (ROS) levels in OPM-2 and KMS-11 cells after SDE2 knockdown. Red fluorescence represents the non-oxidized state, while green fluorescence reflects the oxidized state, indicating lipid peroxidation levels. (D) Bar graph quantifying BODIPY C11 fluorescence intensity in OPM-2 and KMS-11 cells. (E) Cell viability measured by CCK-8 assay in OPM-2 and KMS-11 cells with SDE2 knockdown treated with increasing doses of Erastin (0, 2, 4, 6, and 8 μM) for 24 h. (F) Western blot analysis of ferroptosis-related proteins (NCOA4, ACSL4, FTH1, and GPX4) in H929 and RPMI8226 cells with or without SDE2 overexpression. (G) BODIPY C11 probe staining visualizing lipid peroxidation levels in H929 and RPMI8226 cells with or without SDE2 overexpression. (H) Bar graph quantifying BODIPY C11 fluorescence intensity in H929 and RPMI8226 cells. (I) Cell viability measured by CCK-8 assay in H929 and RPMI8226 cells with SDE2 overexpression treated with increasing doses of Erastin (0, 2, 4, 6, and 8 μM) for 24 h. (J) Biochemical analysis of ferroptosis-related markers (Fe^2+^, MDA, and GSH) in H929 and RPMI8226 cells with SDE2 overexpression. ∗P < 0.05; ∗∗P < 0.01; ∗∗∗P < 0.001.

To investigate the potential involvement of SDE2 in ferroptosis regulation, we examined a series of proteins associated with ferritinophagy and ferroptosis, following SDE2 knockdown. Our results revealed that SDE2 knockdown led to an increase in Nuclear Receptor Coactivator 4 (NCOA4), a key promoter of ferritinophagy, and long-chain-fatty-acid-CoA ligase 4 (ACSL4), which is involved in lipid peroxidation. Concurrently, we observed a decrease in Ferritin Heavy Chain 1 (FTH1), a crucial iron storage and regulatory protein, as well as a reduction in Glutathione Peroxidase 4 (GPX4), an essential antioxidant factor (Fig. 4A). These changes were accompanied by a significant increase in Fe^2+^ levels and malondialdehyde (MDA), a representative lipid peroxidation product, and a marked reduction in glutathione (GSH), a cofactor required for GPX4 function (Fig. 4B).

BODIPY 581/591 C11, a fluorescence probe for detecting lipid peroxidation, exhibits red fluorescence at 591 nm (Ex = 581 nm). Upon oxidation by ROS, the emission peak shifts to 510 nm (Ex = 500 nm), resulting in green fluorescence. Our experiments revealed a substantial increase in the green fluorescence of BODIPY 581/591 C11 in OPM-2 and KMS-11 cells following SDE2 knockdown, indicating heightened oxidative stress and lipid peroxidation (Fig. 4C and D).

To further explore the sensitivity of SDE2-depleted cells to ferroptosis, we treated MM cells with varying concentrations of Erastin (2–8 μM) for 24 h, followed by CCK-8 assays. The results showed that SDE2 knockdown significantly decreased cell viability upon Erastin treatment, suggesting that SDE2 depletion enhances MM cell susceptibility to ferroptosis induction (Fig. 4E).

Conversely, SDE2 overexpression in H929 and RPMI8226 cells led to a decrease in NCOA4 expression, followed by reduced ACSL4 levels and lower MDA production. Additionally, FTH1 expression was upregulated, resulting in reduced free Fe^2+^ levels, and GPX4 and its cofactor GSH were significantly increased (Fig. 4F–J). Consistent with these findings, BODIPY 581/591 C11 staining showed a reduction in green fluorescence, indicating lower levels of lipid peroxidation in SDE2-overexpressing cells (Fig. 4G and H), which corroborated with the reduced MDA synthesis.

Finally, SDE2 overexpression conferred resistance to ferroptosis induction, as evidenced by a decrease in cell death and an increase in survival upon treatment with ferroptosis inducers (Fig. 4I). Collectively, these results suggest that SDE2, through the ATG5-mediated autophagy pathway, negatively regulates ferroptosis levels in MM cells, conferring a protective effect against ferroptosis induction.

### 3.5 The UBL domain of SDE2 is essential for its regulation of autophagy-dependent ferroptosis in MM cells

**Fig. 5:**
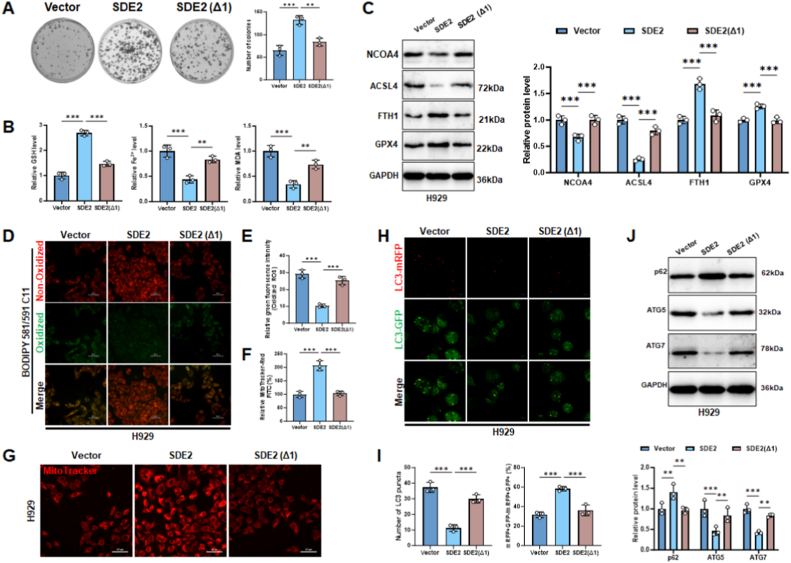
SDE2 promotes MM cell proliferation via its UBL domain–dependent inhibition of ferroptosis and autophagy. (A) Colony formation assays were performed in H929 cells transfected with empty vector, SDE2, or SDE2Δ1. Representative images (left) and quantification of colony numbers (right) are shown. (B) Intracellular glutathione (GSH), ferrous iron (Fe^2+^), and malondialdehyde (MDA) levels were measured using corresponding colorimetric kits. (C) Western blot analysis of ferroptosis-related proteins (NCOA4, ACSL4, FTH1, and GPX4) in the indicated groups; GAPDH was used as a loading control. Densitometric quantification is shown on the right. (D) Lipid peroxidation was assessed by BODIPY 581/591 C11 staining in H929 cells expressing vector, SDE2, or SDE2Δ1. Representative fluorescence images of oxidized (green) and non-oxidized (red) signals are shown. (E) Quantification of oxidized BODIPY fluorescence intensity from (D). (F) Quantification of MitoTracker Red fluorescence intensity in the indicated groups. (G) Mitochondrial morphology was visualized by MitoTracker Red staining in H929 cells. (H) Autophagic flux was evaluated using the mRFP-GFP-LC3 tandem fluorescent-tagged reporter system. Representative images show red (mRFP), green (GFP), and merged puncta. (I) Quantification of LC3 puncta number and percentage of mRFP^+^/GFP^−^ autolysosomes per cell from (H). (J) Western blot analysis of autophagy-related proteins p62, ATG5, and ATG7 in the indicated groups. GAPDH served as a loading control. Densitometric quantification is shown below. ∗P < 0.05; ∗∗P < 0.01; ∗∗∗P < 0.001.

To explore the domain-specific role of SDE2 in multiple myeloma (MM), we compared the effects of full-length SDE2 and its UBL domain–deficient mutant (SDE2Δ1) in H929 cells. Clonogenic assays revealed that SDE2 overexpression markedly enhanced the colony-forming ability of MM cells, whereas this proliferative advantage was completely lost in the Δ1 mutant group (Fig. 5A). This observation suggested that the UBL domain is functionally required for SDE2-driven tumor growth.

Biochemical analysis showed that SDE2 overexpression significantly elevated intracellular GSH levels while reducing Fe^2+^ and MDA accumulation—indicative of ferroptosis suppression. Notably, these metabolic changes were not recapitulated by the SDE2Δ1 mutant (Fig. 5B). In line with this, key ferroptosis regulators such as NCOA4 and ACSL4 were downregulated, while FTH1 and GPX4 were upregulated upon SDE2 expression; however, these effects were abrogated in the Δ1 mutant group (Fig. 5C).

To further assess lipid peroxidation, we employed BODIPY 581/591 C11 staining and observed decreased lipid ROS accumulation in SDE2-overexpressing cells, whereas Δ1-expressing cells maintained high levels of oxidative stress (Fig. 5D and E). Mitochondrial integrity analysis using MitoTracker revealed that SDE2 preserved mitochondrial morphology and reduced fragmentation, in contrast to the Δ1 mutant which failed to confer such protection (Fig. 5F and G).

We next asked whether the autophagy-inhibitory function of SDE2 requires its UBL domain. Imaging of mRFP-GFP-LC3 revealed that SDE2 overexpression decreased autophagosome number and suppressed autophagic flux, as evidenced by reduced LC3 puncta and lower mRFP^+^/GFP^−^ ratio. Again, the Δ1 mutant lost this inhibitory effect (Fig. 5H and I). Immunoblotting confirmed that SDE2 reduced ATG5 and ATG7 expression while increasing p62 levels, consistent with autophagy blockade-an effect not observed with the Δ1 mutant (Fig. 5J).

Thus, these results demonstrate that SDE2 exerts a tumor-promoting effect by coordinately suppressing ferroptosis and autophagy in MM cells, and that the UBL domain is indispensable for mediating these oncogenic functions.

### 3.6 Crosstalk between SDE2-Mediated ferroptosis and autophagy

**Fig. 6:**
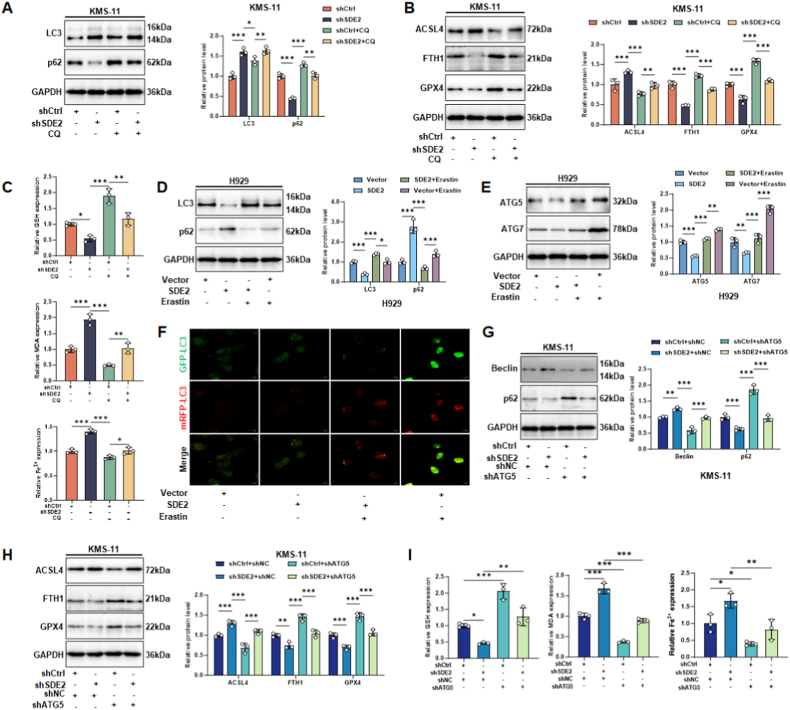
Modulation of autophagy and ferroptosis in MM cells through SDE2 manipulation and pharmacological interventions. KMS-11 cells treated with SDE2 knockdown and/or the autophagy inhibitor chloroquine (CQ): (A) Western blot analysis of autophagy-related proteins LC3 and p62 in KMS-11 cells. Densitometric quantification is shown on the right. (B) Western blot analysis of ACSL4, FTH1, and GPX4 in KMS-11 cells under the same treatments as in (A), with corresponding quantification on the right. (C) Biochemical analysis of ferroptosis-related markers GSH, MDA, and Fe^2+^ in KMS-11 cells. H929 cells treated with SDE2 overexpression and/or the ferroptosis inducer Erastin: (D) Western blot analysis of autophagy-related proteins LC3 and p62 in H929 cells. Densitometric quantification is shown on the right. (E) Western blot analysis and quantification of ATG5 and ATG7 in H929 cells under the same treatments as in (D). (F) mRFP-GFP-LC3 dual fluorescence labeling visualizing autophagic flux in H929 cells, showing autophagosomes (yellow puncta: red + green) and autolysosomes (red-only puncta). KMS-11 cells treated with SDE2 knockdown and/or ATG5 knockdown: (G) Western blot analysis and quantification of Beclin1 and p62 in KMS-11 cells expressing shCtrl or shSDE2, with or without ATG5 knockdown. (H) Western blot analysis of ACSL4, FTH1, and GPX4 in KMS-11 cells with single or combined knockdown of SDE2 and ATG5. Quantification is shown on the right. (I) Biochemical analysis of ferroptosis-related markers GSH, MDA, and Fe^2+^ in KMS-11 cells. ∗P < 0.05; ∗∗P < 0.01; ∗∗∗P < 0.001.

Our work elucidated that SDE2 can regulate ferroptosis through the autophagy pathway, prompting an investigation into the reciprocal relationship between SDE2-mediated autophagy and ferroptosis. In KMS-11 cells, SDE2 knockdown resulted in increased LC3 levels and decreased P62 expression, indicating enhanced autophagy. Subsequent treatment with the autophagy inhibitor chloroquine (CQ) significantly suppressed these changes, effectively blocking autophagy in KMS-11 cells (Fig. 6A). Regarding ferroptosis, CQ treatment prevented the upregulation of ACSL4, malondialdehyde (MDA), and Fe^2+^, as well as the downregulation of Ferritin Heavy Chain 1 (FTH1), while slightly elevating Glutathione Peroxidase 4 (GPX4) and glutathione (GSH) levels compared to the control group (Fig. 6B and C). These findings confirm that SDE2-mediated ferroptosis is autophagy-dependent [29].

Conversely, in H929 cells, overexpression of SDE2 led to decreased LC3 and ATG5 levels, and increased P62 expression, indicating suppressed autophagy and impaired autophagosome-lysosome fusion. Upon treatment with the ferroptosis inducer Erastin, LC3 levels increased, P62 decreased, and ATG5 and ATG7 were dramatically upregulated, suggesting that Erastin promotes autophagy in H929 cells (Fig. 6D and E). Dual-luciferase reporter gene imaging further supported these findings, showing that Erastin treatment accelerated autophagosome maturation and increased the turnover rate of the entire autophagy cycle (Fig. 6F).

In KMS-11 cells, ATG5 knockdown led to decreased Beclin and increased P62 levels, indicating that ATG5 is essential for the autophagic process. The absence of ATG5 inhibited autophagy (Fig. 6G). Regarding ferroptosis, ATG5 knockdown partially counteracted the ferroptosis activation induced by SDE2 knockdown (Fig. 6H and I), suggesting a negative regulatory relationship between SDE2 and ATG5. These results point to a shared downstream autophagy-dependent ferroptosis cascade between SDE2 and ATG5. In other words, the SDE2-ATG5 axis regulates autophagy-dependent ferroptosis in MM cells.

These findings align with previous research, indicating that autophagy and ferroptosis can reinforce each other in MM cells. For instance, FTY720 has been shown to induce both ferroptosis and autophagy via the PP2A/AMPK pathway in MM cells, with autophagy inhibition attenuating ferroptosis, suggesting a positive feedback loop between the two processes [30]. Additionally, studies have demonstrated that ferroptosis-related pathways are involved in MM tumorigenesis and progression, highlighting the importance of understanding the interplay between autophagy and ferroptosis for the development of effective therapeutic strategies.

In summary, our study revealed that SDE2 negatively regulates autophagy and ferroptosis in MM cells through the ATG5-mediated autophagy pathway. Targeting the SDE2-ATG5 axis may offer a novel therapeutic approach to modulate autophagy-dependent ferroptosis in MM, potentially overcoming current treatment limitations and improving patient outcomes.

### 3.7 Opposing regulatory roles of ATG5 and SDE2 in multiple myeloma

**Fig. 7:**
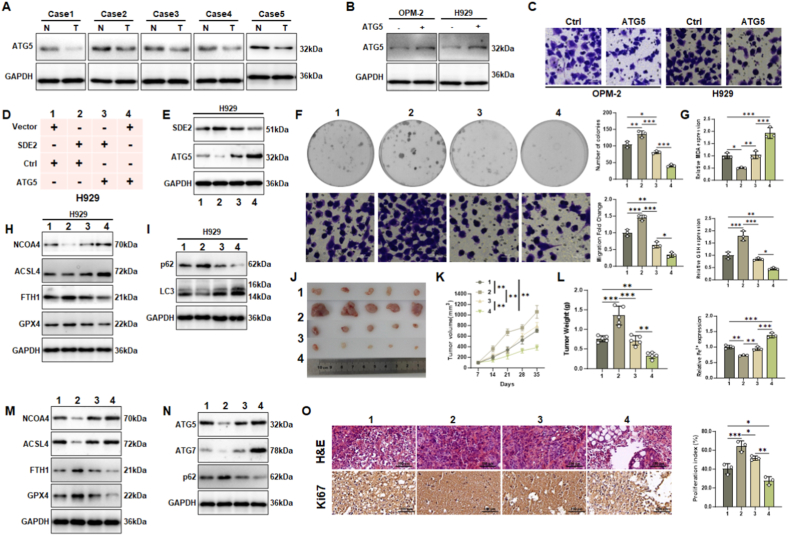
Functional interplay between ATG5 and SDE2 in MM progression and ferroptosis regulation. (A) Western blot analysis of ATG5 expression in malignant cells (T) and non-malignant bone marrow cells (N) isolated from the peripheral blood of MM patients. (B) Western blot analysis confirming the efficiency of ATG5 overexpression in OPM-2 and H929 cells transfected with a plasmid encoding the full-length ATG5 sequence. (C) Crystal violet staining of Transwell migration assays comparing the migratory ability of OPM-2 and H929 cells with or without ATG5 overexpression. (D) Experimental setup for H929 cells treated with SDE2 overexpression, ATG5 overexpression, or a combination of both. (E) Western blot analysis of SDE2 and ATG5 protein levels in H929 cells from the four groups defined in (D). GAPDH was used as a loading control. (F) (Top) Colony formation assay evaluating the proliferative capacity of H929 cells under different treatments. (Bottom) Transwell assay assessing the migratory ability of H929 cells under different treatments. (G) Biochemical analysis of ferroptosis-related markers (MDA, GSH, and Fe^2+^) in H929 cells subjected to different treatments. (H) Western blot analysis of ferroptosis-related proteins (NCOA4, ACSL4, FTH1, and GPX4) in H929 cells under different treatments. (I) Western blot analysis showing the effects of different treatments on LC3 expression and p62 degradation in H929 cells. (J) Representative images of tumors formed 35 days after subcutaneous inoculation of differently treated H929 stable cell lines into nude mice. (K) Tumor growth curves showing volume changes of tumors derived from differently treated H929 cell lines over time. (L) Average tumor weights of tumors derived from differently treated H929 cell lines. (M) Western blot analysis of NCOA4, ACSL4, FTH1, and GPX4 expression levels in tumors derived from differently treated H929 cell lines. (N) Western blot analysis showing the activation levels of the ATG5/ATG7 pathway and p62 degradation in tumors derived from differently treated H929 cell lines. (O) Representative images of H&E staining and Ki67 immunohistochemical staining of tumor sections derived from differently treated H929 cell lines. The bar graph quantifies the percentage of proliferating cells (Ki67-positive). ∗P < 0.05; ∗∗P < 0.01; ∗∗∗P < 0.001.

To further elucidate the roles of ATG5 and SDE2 in MM, we investigated ATG5 expression in clinical samples. Analysis of patient blood samples revealed that ATG5 levels were significantly lower in tumor B cells compared to normal bone marrow cells (Fig. 7A). Overexpression of ATG5 in OPM-2 and H929 cells for 24 h resulted in reduced cell density, as demonstrated by crystal violet staining (Fig. 7B and C). Notably, knockdown of ATG5 in MM cells significantly attenuated ferroptosis, phenocopying the effect of SDE2 overexpression (Fig. S2A–C), suggesting a convergent role for SDE2 and ATG5 loss in ferroptosis resistance.

To explore the functional interplay between SDE2 and ATG5, cells were divided into four groups: 1. control, 2. SDE2 overexpression, and 3. co-overexpression of SDE2 and ATG5 and 4. ATG5 overexpression (Fig. 7D–E). Consistent with previous findings, SDE2 overexpression enhanced H929 cell proliferation in soft agar and migration in Transwell assays. However, these effects were counteracted by simultaneous ATG5 overexpression (Fig. 7F). In the ferroptosis landscape, ATG5 overexpression increased ACSL4 levels and malondialdehyde (MDA) production while reducing FTH1, GPX4, and GSH levels, indicating activation of the ferroptosis cascade (Fig. 7G and H). In the autophagy landscape, ATG5 overexpression elevated LC3 levels and reduced p62 accumulation, suggesting enhanced autophagy activation (Fig. 7I).

H929 cells stably transfected with the aforementioned constructs were subcutaneously injected into immunodeficient mice to generate tumors (Fig. 7J). SDE2 overexpression resulted in the fastest tumor growth and the largest tumor mass, whereas ATG5 overexpression significantly slowed tumor growth and produced the smallest tumor mass (Fig. 7K and L). The co-overexpression of SDE2 and ATG5 yielded an intermediate phenotype, indicating a counterbalancing effect. Western blot analysis of tumor tissues confirmed the findings from *in vitro* experiments, with ferroptosis and autophagy markers showing consistent trends (Fig. 7M and N).

Histopathological examination via H&E staining revealed that SDE2 overexpression led to denser tumor tissue, which was indicative of active cell proliferation. In contrast, ATG5 overexpression resulted in looser tissue structure and vacuolation, suggesting increased cell death (Fig. 7O). Immunohistochemical staining for Ki67, a marker of proliferating cells, showed that SDE2 overexpression maintained nearly all tumor cells in a proliferative state, while ATG5 overexpression reduced the proportion of Ki67-positive cells (Fig. 7O). These findings suggest that SDE2 and ATG5 exert opposing effects on cell cycle regulation through autophagy-dependent ferroptosis pathways.

In parallel knockdown experiments, cells were divided into four groups: a. control, b. SDE2 knockdown, c. ATG5 knockdown, and d, dual knockdown of SDE2 and ATG5 (Fig. S3A–B). SDE2 knockdown decreased GSH levels and increased Fe^2+^ and MDA levels, indicating enhanced ferroptosis. Conversely, ATG5 knockdown increased GSH levels and decreased Fe^2+^ and MDA levels, suggesting increased resistance to ferroptosis in KMS-11 cells (Fig. S3C). In addition, ATG5 knockdown significantly enhanced cell proliferation and migration (Fig. S3D and E).

Surprisingly, the dual knockdown of SDE2 and ATG5 revealed that ATG5 knockdown partially counteracted the ferroptosis-promoting effects of SDE2 knockdown. However, ATG5 knockdown failed to rescue the impaired cell migration caused by SDE2 knockdown, suggesting that SDE2 may regulate additional downstream targets beyond ATG5, which critically influences cell migration independent of the ferroptosis pathway (Fig. S3E).

These results demonstrate that ATG5 and SDE2 exhibit opposing regulatory roles in MM, with ATG5 promoting ferroptosis and autophagy while SDE2 enhances proliferation and migration. The interplay between these two proteins highlights their potential as therapeutic targets in MM.

### 3.8 SDE2 knockdown combined with autophagy induction exerts synergistic anti-tumor effects

**Fig. 8:**
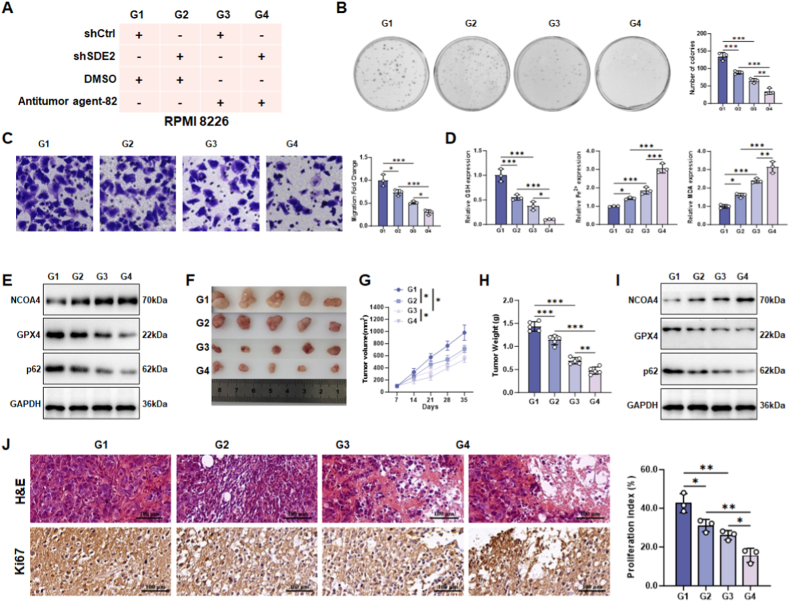
Effects of SDE2 knockdown and Antitumor agent-82 treatment on RPMI 8226 cells *in vitro* and *in vivo*. (A) Experimental setup for RPMI 8226 cells treated with SDE2 knockdown, Antitumor agent-82, or a combination of both. (B–C) (B) Colony formation assay evaluating the proliferative capacity of RPMI 8226 cells under different treatments. (C) Transwell migration assay assessing the migratory ability of RPMI 8226 cells under different treatments. (D) Biochemical analysis of ferroptosis-related markers (GSH, Fe^2+^, and MDA) in RPMI 8226 cells under different treatments. (E) Western blot analysis of ferroptosis-related proteins (NCOA4, ACSL4, FTH1, and GPX4) in RPMI 8226 cells subjected to different treatments. (F) Representative images of tumors formed 35 days after subcutaneous inoculation of differently treated RPMI 8226 stable cell lines into nude mice (n = 5). (G) Tumor growth curves showing volume changes of tumors derived from differently treated RPMI 8226 cell lines over time (n = 5). (H) Tumor weight was recorded at the endpoint (Day 35) for each group (n = 5). (I) Western blot analysis of NCOA4, GPX4, and p62 expression levels in tumors derived from differently treated RPMI 8226 cell lines (n = 5). (J) Representative images of H&E staining and Ki67 immunohistochemical staining of tumor sections derived from RPMI 8226 cell lines. The bar graph quantifies the percentage of proliferating cells (Ki67-positive) (n = 5). ∗P < 0.05; ∗∗P < 0.01; ∗∗∗P < 0.001.

In this study, we explored the therapeutic potential of combining SDE2 knockdown with pharmacological autophagy induction in MM cells. RPMI 8226 cells were categorized into four groups: G1. control, G2. SDE2 knockdown, G3. treatment with Antitumor agent-82, and G4. SDE2 knockdown combined with Antitumor agent-82 treatment (Fig. 8A). Antitumor agent-82 is recognized for its ability to induce autophagy via the ATG5/ATG7 signaling pathway [31].

Our results demonstrated that SDE2 knockdown alone significantly inhibited proliferation and migration of RPMI 8226 cells. While Antitumor agent-82 treatment exhibited slightly superior effects compared to SDE2 knockdown, the combination of both treatments resulted in a markedly enhanced anti-tumor effect (Fig. 8B–C).

Biochemical assays revealed that the combined treatment progressively upregulated the ferroptosis marker NCOA4, accompanied by changes in GPX4 and p62 levels, indicating enhanced ferroptosis and autophagy (Fig. 8D). Additionally, there was a gradual decrease in glutathione (GSH) levels, alongside increased Fe^2+^ release and malondialdehyde (MDA) production, further confirming the activation of ferroptosis (Fig. 8E).

To validate these findings *in vivo*, RPMI 8226 cells, with or without stable SDE2 knockdown, were subcutaneously transplanted into immunodeficient nude mice. After one week, the mice received daily intraperitoneal injections of Antitumor agent-82 or vehicle control for 35 days (Fig. 8F). Tumor growth was significantly slowed in the treatment groups, with the combination group showing the most pronounced reduction in tumor size and weight (Fig. 8G and H). Western blot analysis of tumor tissues revealed trends consistent with the *in vitro* results, demonstrating that SDE2 knockdown and/or Antitumor agent-82 treatment synergistically enhanced ferroptosis and autophagy (Fig. 8I).

Histopathological examination via H&E staining showed a progressive decrease in tissue density and increased vacuolation in the treatment groups. Additionally, Ki67 staining revealed a gradual reduction in Ki67-positive cells, indicating that targeting SDE2 combined with pharmacological autophagy induction effectively disrupted the cell cycle of MM cells, thereby suppressing tumorigenesis (Fig. 8J).

These findings suggest that the combination of SDE2 knockdown and Antitumor agent-82 treatment synergistically enhances ferroptosis and autophagy, leading to significant anti-tumor effects both *in vitro* and *in vivo*. This approach represents a promising therapeutic strategy for treating MM.

## 4 Discussion

This study systematically investigated the oncogenic role of SDE2 in MM, uncovering its mechanistic link with autophagy suppression and ferroptosis resistance. Through bioinformatics analysis of clinical datasets and functional validation in MM cell lines and xenograft models, we demonstrated that SDE2 is overexpressed in MM and correlates with poor prognosis. Mechanistically, SDE2 binds to ATG5, a core autophagy protein, and promotes K48-linked ubiquitination and proteasomal degradation. The SDE2-ATG5 axis disrupts autophagy flux, stabilizes antioxidant systems (e.g., GPX4/GSH), and reduces intracellular iron accumulation, thereby shielding MM cells from ferroptosis. Genetic or pharmacological inhibition of SDE2 restores ATG5 levels, reactivates autophagy, and sensitizes MM cells to ferroptosis inducer, as evidenced by elevated lipid peroxidation and Fe^2+^ levels. *In vivo*, the combined targeting of SDE2 and pharmacological activation of the ATG5/7 pathway synergistically suppresses tumor growth. These findings establish the SDE2-ATG5 axis as a pivotal regulator of the autophagy-ferroptosis crosstalk in MM.

Our work aligns with growing evidence that autophagy and ferroptosis are interconnected yet context-dependent processes in cancer biology [23]. Although basal autophagy is generally pro-survival, it can trigger ferroptosis in a cargo-dependent manner through mechanisms such as iron overload and lipid peroxidation, a phenomenon observed in cancers such as pancreatic adenocarcinoma and hepatocellular carcinoma [[32], [33], [34]]. The role of ATG5 in this balance has been documented [35], but upstream regulators controlling its stability in MM remain unexplored. Our identification of SDE2 as a ubiquitin-like protein driving ATG5 degradation fills this gap, extending our understanding of how proteasomal pathways modulate sensitivity to cell death. This finding contrasts with prior studies implicating SDE2 in DNA repair and RNA splicing in solid tumors, highlighting its context-dependent oncogenic functions [24,36].

Furthermore, our findings align with emerging evidence of the critical role of ubiquitination in regulating ferroptosis. For example, the E3 ligase TRIM26 inhibits ferroptosis by catalyzing K63-linked ubiquitination of GPX4 in glioma [37]. Similarly, Imatinib induced ferroptosis in gastrointestinal stromal tumors by promoting STUB1-mediated ubiquitination of GPX4 [38]. In non-small cell lung cancer, TRIM3 enhances ferroptosis by facilitating K11-linked ubiquitination and degradation of SLC7A11/xCT [39]. In this study, we identify a distinct mechanism in which SDE2-mediated ubiquitination of ATG5 indirectly stabilizes NCOA4, suppressing autophagy-dependent iron release and thereby enhancing the antioxidant capacity of cells. This regulatory axis broadens the therapeutic landscape for MM, which has traditionally relied on proteasome inhibitors, such as bortezomib. Notably, SDE2 lacks a canonical E3 ligase domain, suggesting it may function as a scaffold to recruit yet unidentified E3 ligases. The UBL domain of SDE2 appears essential for this process, as its deletion abolishes ATG5 ubiquitination and degradation. This raises the possibility that the UBL domain may serve as an interface to interact with endogenous E3 ligases or components of the ubiquitination machinery. Future work will aim to map the minimal interacting domains, identify E3 ligases that partner with SDE2, and dissect whether SDE2 serves a direct catalytic or adaptor role in ATG5 ubiquitination. These studies will help clarify the regulatory axis of SDE2–ATG5 and its relevance in autophagy and ferroptosis control in MM.

Compared to previous studies, our work offers several distinct advantages. First, we provided comprehensive evidence linking SDE2 expression to poor MM prognosis by combining bioinformatics, clinical data, and experimental validation. Second, we identified a novel mechanism through which SDE2 regulates autophagy and ferroptosis via ATG5 ubiquitination, thereby advancing our understanding of ubiquitin-like proteins in cancer biology. Third, our study demonstrated the therapeutic potential of targeting SDE2 in combination with autophagy inducers, offering a promising strategy to enhance ferroptosis activation and overcome drug resistance in MM. These findings not only provide mechanistic insights but also have direct translational relevance, paving the way for future therapeutic interventions.

However, this study has some limitations. First, the reliance on established MM cell lines may not fully capture the genetic and epigenetic heterogeneity of patient tumors. Future studies should validate these findings in primary MM cells and their diverse molecular subtypes. Second, while we identified the UBL domain of SDE2 as critical for ATG5 ubiquitination, the specific E3 ligase facilitating this interaction remains unknown. Elucidation of this intermediate can refine therapeutic targeting. Third, the long-term safety of combined SDE2-ATG5 inhibition requires scrutiny, given the essential role of autophagy in normal cellular homeostasis. Finally, our *in vivo* subcutaneous transplantation models did not fully replicate the complexity of the bone marrow microenvironment, which may influence MM cell behavior.

Our findings redefine MM as a malignancy driven not only by genetic mutations, but also by dysregulated post-translational modifications (PTMs) that govern proteostasis and cell death. The SDE2-ATG5 axis exemplifies how ubiquitination rewires cellular adaptability, enabling MM cells to evade both therapy-induced stress and endogenous death pathways. This paradigm shift underscores the potential of PTM-targeted therapies for hematologic malignancies, complementing the existing genomic approaches.

Beyond MM, our findings encourage investigating pathologies involving iron dysregulation or immune dysregulation. For example, autoreactive plasma cells drive disease persistence in SLE. Targeting the autophagy-ferroptosis crosstalk in these cells could provide a novel therapeutic strategy. However, rigorous preclinical validation is essential for efficacy and safety. Are SLE plasma cells similarly dependent on SDE2-ATG5 signaling? Does induced ferroptosis further exacerbate systemic inflammation [40]?

In summary, this study revealed that the SDE2-ATG5 axis is a linchpin of autophagy-ferroptosis crosstalk in MM. Despite the limitations, our work lays a foundation for precision therapies targeting ubiquitination networks and iron metabolism. The speculative extension to immune-related pathologies, although scientifically plausible, requires further investigation to assess its feasibility and risks. Ultimately, these findings highlight the transformative potential of mechanistically informed, PTM-focused therapies in oncology and beyond.

## Ethics approval and consent to participate

All human samples were obtained with informed consent under protocols approved by the Institutional Review Board of Ruijin Hospital Affiliated to Shanghai Jiao Tong University School of Medicine. Animal experiments were conducted in accordance with the guidelines of the Animal Care and Use Committee of Ruijin Hospital Affiliated to Shanghai Jiao Tong University School of Medicine.

## Consent for publication

Not applicable.

## Funding

This study was supported by the University 10.13039/501100003995Natural Science Foundation of Anhui Province (2023AH053319).

## CRediT authorship contribution statement

**Liang Xia:** Data curation, Formal analysis, Funding acquisition, Methodology, Validation, Visualization, Writing – original draft. **Jing Bao:** Data curation, Formal analysis, Methodology, Software, Supervision, Validation. **Xiao-wen Chen:** Data curation, Investigation, Methodology, Resources, Visualization. **Yu-Chen Zhao:** Resources, Software, Supervision, Validation, Visualization, Writing – review & editing. **Xiang Wang:** Methodology, Software, Supervision, Validation. **Yu Zheng:** Conceptualization, Investigation, Methodology, Project administration, Resources, Software, Supervision, Validation, Visualization, Writing – review & editing.

## Declaration of competing interest

The authors declare the following financial interests/personal relationships which may be considered as potential competing interests: Liang Xia reports financial support was provided by University Natural Science Foundation of Anhui Province. If there are other authors, they declare that they have no known competing financial interests or personal relationships that could have appeared to influence the work reported in this paper.

## Data Availability

The data analyzed during the current study are available from the corresponding author on reasonable request.
