# Supplementary material for: Inhibition of SDE2 promotes autophagy-dependent ferroptosis in multiple myeloma
Source: Redox Biol. 2026 Jan 16;91:104007. doi: 10.1016/j.redox.2026.104007 (PMC12907901; doi:10.1016/j.redox.2026.104007)
Supplement: Multimedia component 1 [file mmc1.docx]

**Supplementary**

| **Figure S1: Effects of SDE2 overexpression on MM cell behavior and autophagy.** |
| --- |
| **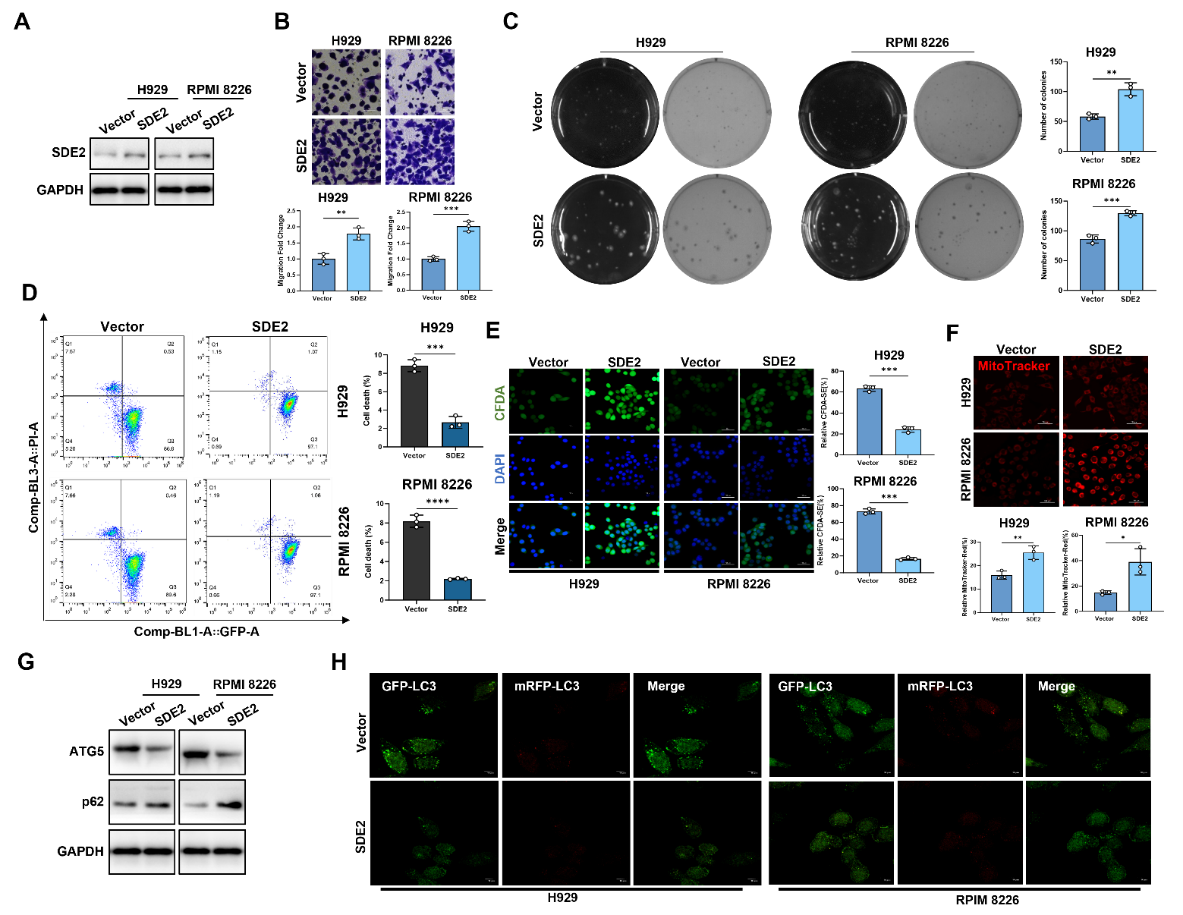** |
| (A) Western blot analysis confirming the efficiency of SDE2 overexpression in H929 and RPMI 8226 cells transfected with a plasmid containing the full-length SDE2 sequence. (B) Crystal violet staining of Transwell migration assays comparing the migratory potential of H929 and RPMI 8226 cells with or without SDE2 overexpression (top). The bar graph quantifies fold changes in migration (bottom). (C) Representative images of soft agar colony formation assays under epi-illumination and transmitted light (left). The bar graph quantifies the number of colonies formed (right).  (D) Flow cytometry analysis of PI/CFDA-stained H929 and RPMI 8226 cells with or without SDE2 overexpression. Q2 indicates dead cells, and Q4 indicates live cells (left). The bar graph shows the percentage of dead cells (right). (E) Fluorescence imaging of CFDA/DAPI-stained H929 and RPMI 8226 cells with or without SDE2 overexpression. (F) MitoTracker staining assessing mitochondrial activity in H929 and RPMI 8226 cells with or without SDE2 overexpression. (G) Western blot analysis showing p62 and ATG5 expression levels in H929 and RPMI 8226 cells with or without SDE2 overexpression. (H) mRFP-GFP-LC3 dual fluorescence labeling visualizing autophagosomes (yellow puncta: red + green) and autolysosomes (red-only puncta) in H929 and RPMI 8226 cells with or without SDE2 overexpression. The bar graph quantifies the total number of puncta and the relative proportion of autophagosomes to autolysosomes. *P < 0.05; **P < 0.01; ***P < 0.001. |

| **Figure S2: Inhibition of ATG5 suppresses ferroptosis in MM cells.** |
| --- |
| 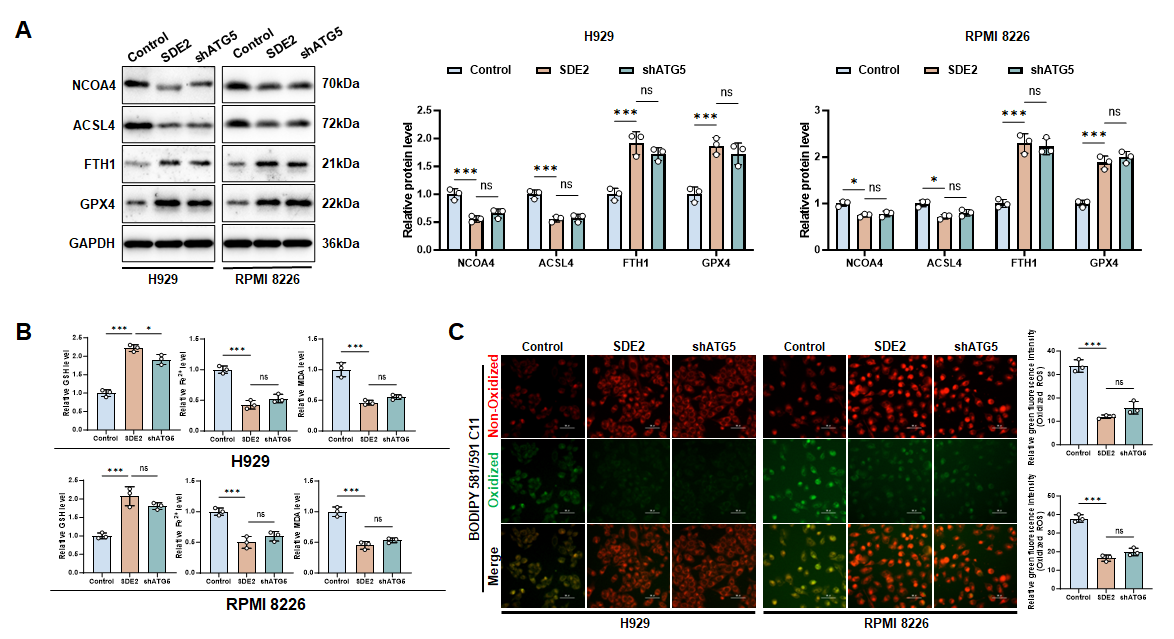 |
| (A) Western blot analysis of NCOA4, ACSL4, FTH1, and GPX4 in H929 and RPMI 8226 cells transduced with control vector, SDE2 overexpression, or shATG5. GAPDH was used as loading control. Densitometric quantification of protein levels is shown on the right.  (B) Flow cytometric analysis of intracellular GSH, Fe²⁺, and MDA levels in the indicated groups of H929 and RPMI 8226 cells.  (C) Representative images of BODIPY 581/591 C11 staining in H929 and RPMI 8226 cells to assess lipid peroxidation. Oxidized (green) and non-oxidized (red) fluorescence signals were merged to visualize lipid ROS distribution. Quantification of green/red ratio is shown on the right. *P < 0.05; **P < 0.01; ***P < 0.001. |

| **Figure S3: Combined effects of SDE2 and ATG5 knockdown on ferroptosis and cellular behavior in MM cells.** |
| --- |
| 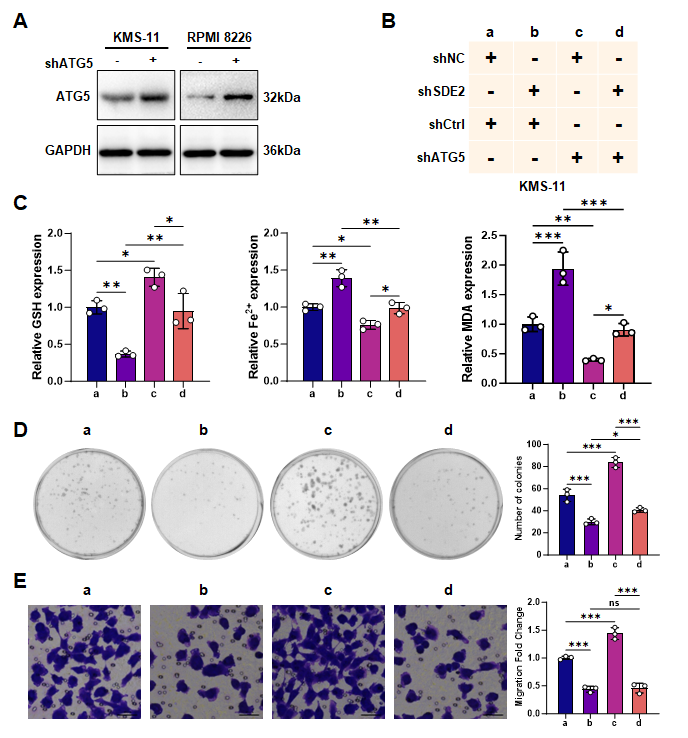 |
| (A) Western blot analysis confirming the efficiency of ATG5 knockdown in KMS-11 and RPMI8226 cells using shATG5. (B) Experimental setup for KMS-11 cells treated with SDE2 knockdown, ATG5 knockdown, or a combination of both. (C) Biochemical analysis of ferroptosis-related markers (MDA, GSH, and Fe²⁺) in KMS-11 cells under different treatment conditions. (D) Colony formation assay evaluating the proliferative capacity of KMS-11 cells under different treatments. (E) Transwell migration assay assessing the migratory ability of KMS-11 cells under different treatments.  *P < 0.05; **P < 0.01; ***P < 0.001. |
